# *Staphylococcus aureus* Causing Skin and Soft Tissue Infections in Companion Animals: Antimicrobial Resistance Profiles and Clonal Lineages

**DOI:** 10.3390/antibiotics11050599

**Published:** 2022-04-29

**Authors:** Sofia Santos Costa, Rute Ribeiro, Maria Serrano, Ketlyn Oliveira, Carolina Ferreira, Marta Leal, Constança Pomba, Isabel Couto

**Affiliations:** 1 Global Health and Tropical Medicine, GHTM, Instituto de Higiene e Medicina Tropical, IHMT, Universidade Nova de Lisboa, UNL, Rua da Junqueira 100, 1349-008 Lisboa, Portugal; scosta@ihmt.unl.pt (S.S.C.); a21000795@ihmt.unl.pt (R.R.); a21000870@ihmt.unl.pt (M.S.); mmm0012@ihmt.unl.pt (K.O.); carolinaf@ihmt.unl.pt (C.F.); a21001253@ihmt.unl.pt (M.L.); 2 CIISA, Centre of Interdisciplinary Research in Animal Health, Faculty of Veterinary Medicine, University of Lisbon, Avenida da Universidade Técnica, 1300-477 Lisboa, Portugal; cpomba@fmv.ulisboa.pt; 3 GeneVet, Laboratório de Diagnóstico Molecular Veterinário, Rua Quinta da Nora Loja 3B, 2790-140 Carnaxide, Portugal

**Keywords:** *Staphylococcus aureus*, MRSA, companion animals, antimicrobial resistance, heavy metals, clonal lineages, One Health

## Abstract

*Staphylococcus aureus* is a relevant agent of skin and soft tissue infections (SSTIs) in animals. Fifty-five *S. aureus* comprising all SSTI-related isolates in companion animals, collected between 1999 and 2018 (Lab 1) or 2017 and 2018 (Lab 2), were characterized regarding susceptibility to antibiotics and heavy metals and carriage of antimicrobial resistance determinants. Clonal lineages were established by PFGE, MLST and *agr* typing. Over half of the isolates (56.4%, 31/55) were methicillin-resistant *S. aureus* (MRSA), and 14.5% showed a multidrug resistance (MDR) phenotype. Resistance was most frequently observed for beta-lactams (81.8%, related to *blaZ* and/or *mecA*), fluoroquinolones (56.4%) and macrolides/lincosamides (14.5%, related to *erm*(A) or *erm*(C)). The distributions of heavy-metal MICs allowed the detection of non-wild-type populations associated with several resistance genes. The collection showed genetic diversity, with prevalence of clonal lineage ST22-*agr*I (45.5%, 25/55), comprising only MRSA isolates, and several less frequently detected clones, including ST5-*agr*II (14.6%, 8/55), ST398-*agr*I (9.1%, 5/55) and ST72-*agr*I (7.3%, 4/55). This work highlights the high frequency of SSTI-related MRSA strains that reflect the clonal lineages circulating both in companion animals and humans in Portugal, reinforcing the need for a One Health approach when studying staphylococci causing infections in companion animals.

## 1. Introduction

Skin and soft tissue infections (SSTIs), particularly pyoderma, are among the most common causes of antimicrobial prescription in veterinary medicine [1]. Coagulase-positive staphylococci are the main bacterial pathogens underlying these infections [2]. In dogs, *Staphylococcus pseudintermedius* accounts for nearly 90% of pyoderma cases, whereas *Staphylococcus aureus* and *Staphylococcus coagulans* are interchangeably the second most common pathogen accounting for up to 10% of pyoderma cases [2,3]. In cats, *S. aureus* is a more frequent agent of pyoderma than *S. pseudintermedius*, although there is less available literature for these cases [4]. The prevalence rate of these bacterial pathogens in other companion animals, such as rabbits, is relatively unknown.

*S. aureus* is a transient colonizer of both cats and dogs and can act as an opportunistic pathogen that causes, besides pyoderma, otitis externa, upper respiratory tract infections, cystitis, abscesses, osteomyelitis or endocarditis [5,6]. In rabbits, it causes skin infections and mastitis [5].

The emergence of antimicrobial-resistant strains amongst these staphylococci is worrisome, limiting the arsenal of antimicrobials available to treat infections. Methicillin-resistant *S. aureus* (MRSA) was first described in humans in 1961 [7]. These strains carry the mobile element staphylococcal cassette chromosome *mec* (SCC*mec*) that harbors the *mecA* gene, which encodes an additional penicillin-binding protein (PBP2a) with a lower binding affinity towards beta-lactam antibiotics [8]. Therefore, these strains are resistant to all beta-lactams antibiotics, except for fifth-generation cephalosporins [8]. The report of the first MRSA strains in animals was associated with bovine infections and dates to the 1970s [9]. The first occurrence of these strains in companion animals was reported in 1988 [10]. Since then, MRSA has been increasingly isolated from infection cases in companion animals, particularly in the last two decades [11]. A study surveying the staphylococci causing several types of infections in pets in Portugal during a 16-year period reported an increasing trend of MRSA as well as an overall frequency rate of 40.7% of these strains [12]. This increasing resistance extended to other relevant antibiotic classes and multidrug resistance (MDR) [12].

In the veterinary field, as in human medicine, heavy metals are used both in therapy (included in formulations used for the treatment of SSTIs) and in the disinfection of devices and surfaces [13,14]. Copper and silver are the most widely used, the latter being the most used for the treatment and prevention of SSTIs [15]. However, cases of reduced susceptibility to these heavy metals have been reported due to their excessive use [15]. The genetic determinants involved in reduced susceptibility to heavy metals (including copper, arsenate and cadmium) are commonly encountered on mobile genetic elements, many of which harbor additional antibiotic resistance genes [16]. As such, heavy metals have been hypothesized as a selective factor toward the dissemination of antimicrobial resistance [16].

The increasing burden of antimicrobial resistance in *S. aureus* and other staphylococci causing skin infections in companion animals is worrisome as it limits the available therapeutic options for the management of these infections, with a direct impact on animal morbidity and mortality.

In the last decades, growing evidence suggests that close contact with humans is a source of transfer of *S. aureus* strains from humans to animals and vice-versa [6], which also provides for the possible movement of antimicrobial resistance determinants between hosts [6,17]. This evidence, together with the increasing presence of companion animals in human households, has led to the consideration of animal-associated MRSA strains as a public health risk [11].

In the present study, we characterized a collection of *S. aureus* associated with SSTIs in companion animals, isolated from 1999 to 2018, in Lisbon, Portugal. We aimed to establish the circulating *S. aureus* clonal lineages associated with SSTIs in these animals as well as their relation with host and antimicrobial resistance traits, including susceptibility levels towards several heavy-metal compounds usually applied in topical therapeutics and disinfection procedures.

## 2. Results

### 2.1. Antibiotic Susceptibility Profiles and Relationship with Resistance Determinants

We determined the susceptibility profiles for a wide panel of antibiotics used in veterinary and/or human medicine (Table 1). Antibiotic resistance was a common feature of the collection, detected in 47 isolates (47/55, 85.5%). Resistance to one or two antibiotic classes was detected for 12 and 27 isolates (21.8% and 49.1%), respectively. Eight isolates (8/55, 14.5%) presented a MDR phenotype.

Resistance to penicillin was detected in 45 (45/55, 81.8%) isolates. More than half of the isolates (31/55, 56.4%) were MRSA (*mecA*^+^ and cefoxitin resistant), but only six out of the 31 MRSA detected were MDR. Resistance to fluoroquinolones was common, detected in 31 isolates (31/55, 56.4%). A single isolate showed an intermediate phenotype towards ciprofloxacin and enrofloxacin and was considered resistant according to CLSI recommendations [18,19]. We have also observed resistance to erythromycin and inducible resistance to clindamycin (14.5%, 8/55); kanamycin, tobramycin and gentamycin (3.6%, 2/55); tetracycline (3.6%, 2/55) and minocycline (1.8%, 1/55); fusidic acid (1.8%, 1/55); and chloramphenicol (1.8%, 1/55). Although no breakpoints are available for bacitracin, one isolate presented no inhibition halo and was considered resistant. All isolates were susceptible to tigecycline, amikacin, rifampicin, trimethoprim-sulfamethoxazole, linezolid, mupirocin and quinupristin-dalfopristin.

Although no breakpoints are available for apramycin and florfenicol, the application of the recently proposed epidemiological cut-off (ECOFF) values for apramycin (ECOFF = 15 mm) and florfenicol (ECOFF = 21 mm) [21], allowed the detection of one non-wild-type isolate (NWT, carrying acquired resistance mechanism(s) phenotypically expressed) towards florfenicol.

Resistance to penicillin, cefoxitin and fluoroquinolones was the most common resistance pattern encountered, showed by 24 out of the 55 isolates (24/55, 43.6%), followed by monoresistance to penicillin, detected in 11 isolates (11/55, 20.0%). MDR phenotypes were diverse, generally represented by resistance to beta-lactams and fluoroquinolones together with erythromycin/clindamycin, tetracyclines or aminoglycosides.

The presence of antibiotic-resistance determinants was assessed for all isolates (*blaZ*, *mecA*) or isolates presenting phenotypic resistance (categorized as resistant, intermediate by CLSI or NWT for florfenicol) (Table 1). The *blaZ* and *mecA* genes were detected in all isolates resistant to penicillin or cefoxitin, respectively. Resistance to macrolides and lincosamides was associated with *erm*(C) or *erm*(A). Resistance to aminoglycosides and fusidic acid was linked to the *aadD* and *fusC* genes, respectively. The resistance genes *tet*(K) and *tet*(M) were found jointly in one isolate displaying a resistant phenotype to tetracycline and intermediate to minocycline. The single isolate that was resistant to chloramphenicol and NWT for florfenicol carried the *fexA* gene.

### 2.2. Detection of Reduced Susceptibility to Heavy Metals

The MIC distributions of five heavy metals were analyzed for the entire collection to estimate the respective cut-off (CO_WT_) values. The estimated CO_WT_ were then used to detect NWT populations, potentially carrying acquired resistance mechanism(s) with phenotypic expression for each heavy metal.

**Figure 1:**
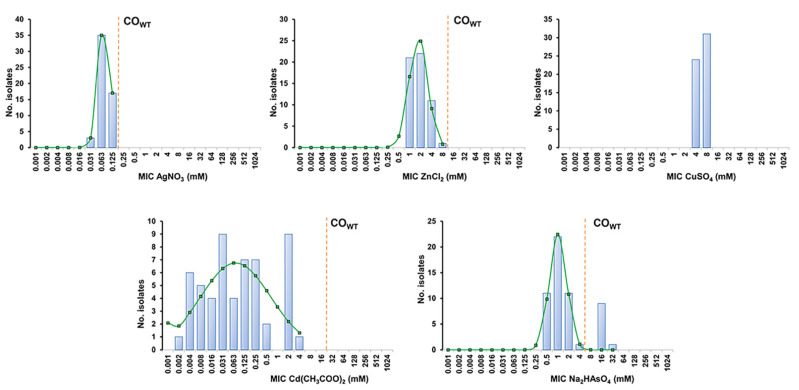
MIC distributions and estimation of cut-off (CO_WT_) values of the 55 SSTI-related *S. aureus* from companion animals for the heavy metals silver nitrate (AgNO_3_), zinc chloride (ZnCl_2_), copper sulphate (CuSO_4_), cadmium acetate (Cd(CH_3_COO)_2_) and disodium hydrogen arsenate (Na_2_HASO_4_). The CO_WT_ values were estimated with the iterative statistical method using the ECOFFinder datasheet available at https://www.eucast.org/mic_distributions_and_ecoffs/ (accessed on 10 November 2021). The blue columns represent the MIC values determined for all isolates, whereas the green line indicates the distribution of MICs for the wild-type (WT) population estimated by the ECOFFinder. The dashed orange line indicates the CO_WT_ value that corresponds to the highest MIC value of the estimated WT population.

The 55 *S. aureus* presented distinct susceptibility levels towards the five heavy metals tested (Figure 1). All isolates were highly susceptible to silver nitrate (MIC range 0.03–0.125 mM) and cadmium acetate (MIC range 0.002–4 mM) and were less susceptible to zinc chloride (MIC range 1–8 mM), copper sulphate (MIC range 4–8 mM) and disodium hydrogen arsenate (MIC range 0.5–32 mM).

The *S. aureus* collection displayed unimodal MIC distributions for silver nitrate, zinc chloride and copper sulphate, encompassing only two to four MIC values (Figure 1 and Table 2). The application of the estimated CO_WT_ values did not reveal NWT populations towards the first two heavy metals. In the case of copper sulphate, the restricted MIC values found did not allow the estimation of a CO_WT_ value by ECOFFinder (which requires a minimum of three distinct MIC values). The MIC distribution for disodium hydrogen arsenate was bimodal, and the estimated CO_WT_ indicates that 10 isolates (10/55, 18.2%) belong to the NWT population and potentially carry resistance determinants towards arsenate (Figure 1 and Table 2). In fact, screening for an arsenate resistance determinant showed that all but one of the NWT isolates harbored the *arsB* gene. The MIC distribution of cadmium acetate was multimodal, suggesting the presence of a significant proportion of populations carrying distinct resistance determinants associated with different susceptibility levels. Although the wide distribution of MIC values for this heavy metal prevented an accurate estimation of the respective CO_WT_ (SD > 1 log_2_) (Table 2), screening of cadmium resistance determinants detected carriage of the *cadA* (9/55, 16.4%) or *cadD* genes (18/55, 32.7%), which were linked, respectively, to high cadmium MICs (2–4 mM) or lower and more variable MICs (0.004–0.5 mM). Of interest, all isolates carrying *cadA* also harbored the arsenate resistance gene *arsB*.

### 2.3. S. aureus Clonal Lineages Associated with SSTIs in Companion Animals

**Figure 2:**
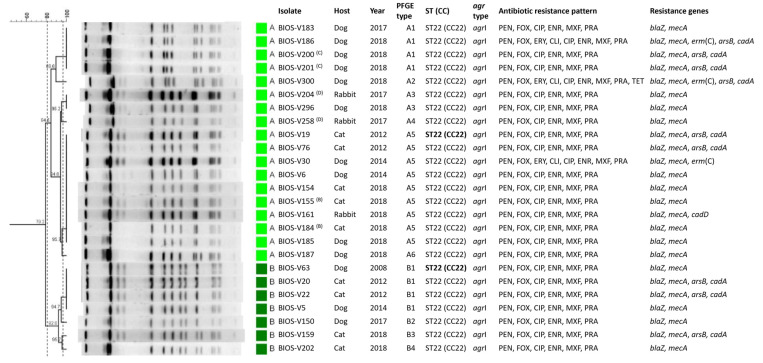

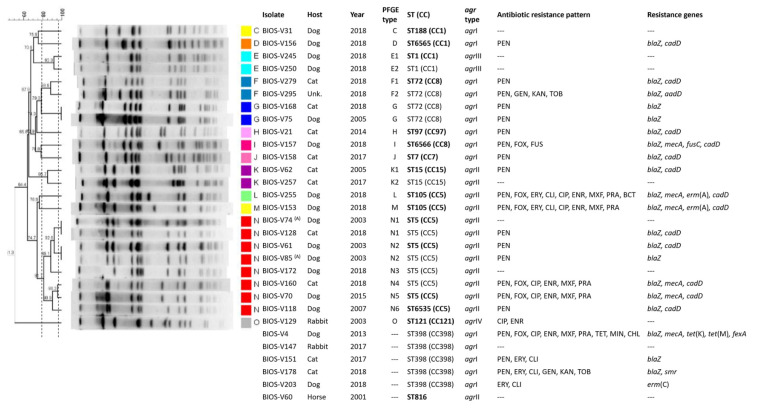
*Sma*I-PFGE profiles of the *S. aureus* isolates associated with SSTIs in companion animals and corresponding clonal lineages as determined by MLST and their correlation with host, *agr* types, phenotypic and genotypic resistance traits. The pairs of isolates recovered from the same animal are marked by (A) to (D), where each letter corresponds to a different animal. The dendrogram was built using Bionumerics and the UPGMA algorithm, using the Dice coefficient, an optimization of 0.5% and a tolerance of band of 1%. The dashed lines correspond to the similarity criteria for considering isolates belonging to the same PFGE type (≥80%) or subtype (≥97%). Isolates sharing the same PFGE type or subtype were considered as belonging to the same sequence type (ST). The isolates subjected to MLST are indicated in bold type. Unk: unknown host; CC: clonal complex; ST: sequence type; PFGE: pulsed-field gel electrophoresis; PEN: penicillin; CXI: cefoxitin; ERY: erythromycin; CLI: clindamycin; CIP: ciprofloxacin; ENR: enrofloxacin; MOX: moxifloxacin; PRA: pradofloxacin; KAN: kanamycin; GEN: gentamycin; TOB: tobramycin; FUS: fusidic acid; BCT: bacitracin.

Characterization of all isolates by *Sma*I-PFGE allowed the assignment of 15 types (A to O) and 22 subtypes; *Sma*I-PFGE types A, N and B were the predominant ones, corresponding to 18 (32.7%), 8 (14.5%) and 7 (12.7%) isolates, respectively (Figure 2). The absence of *Sma*I macrorestriction was observed for six isolates. Simpson’s index of diversity (SID), calculated based upon the PFGE *Sma*I-macrorestriction profiles (including lack of profile), revealed a diverse *S. aureus* population (SID = 0.84, CI: 0.77–0.92).

**Figure 3:**
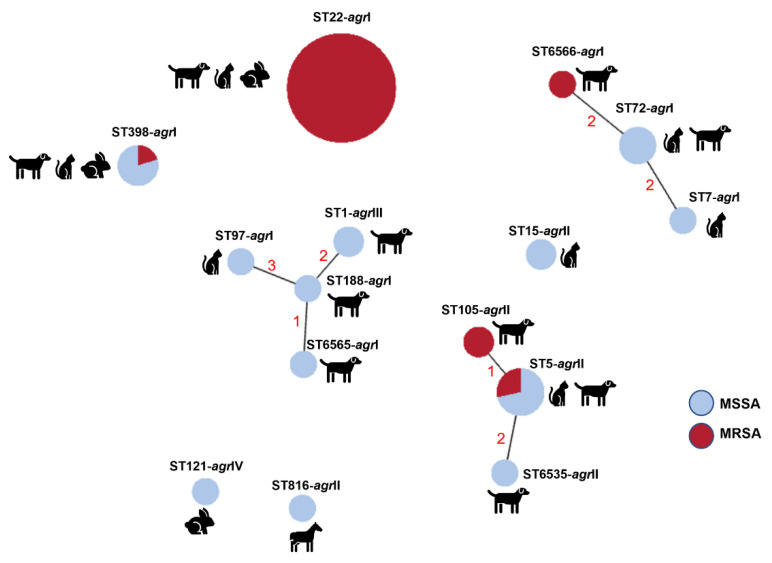
Relationship between clonal lineages, host and methicillin resistance among the 55 SSTI-related *S. aureus* isolates. The relationship between clonal lineages was estimated using the goeBurst algorithm available at PhyloViz Online. Clonal lineages sharing at least four alleles are linked with solid lines; numbers correspond to the diverging alleles.

An isolate representative of each PFGE type was selected for typing by multilocus sequence typing (MLST). Resistance to *Sma*I macrorestriction is a characteristic of isolates belonging to clonal lineage ST398 [22]. We applied a PCR approach [23] to confirm the ST398 genetic lineage of the six isolates resistant to digestion by *Sma*I. Five of those six isolates did belong to ST398, whereas the sixth isolate was further characterized by MLST and classified as the singleton ST816. In total, 15 sequence types (STs) were identified within the collection (Figure 3), representing nine clonal complexes (CCs). The predominant CC was CC22 (ST22), corresponding to nearly half the isolates (25/55, 45.5%), which were assigned to *Sma*I-PFGE types A and B. The CC5 (ST5, ST105 and the newly identified ST6535) was the second most frequent complex (10/55, 18.2%), followed by CC8 (ST72 and the new ST6566) and CC398 (ST398) as the third predominant complexes, each represented by five isolates (5/55, 9.1%). Other CCs identified were CC1 (ST1, ST188 and the new ST6565) represented by four isolates (4/55, 7.3%); CC15 (ST15, 2/55, 3.6%); CC97 (ST97, 1/55, 1.8%); CC7 (ST7, 1/55, 1.8%) and CC121 (ST121, 1/55, 1.8%) (Figure 2).

Typing of *agr* revealed an association between *agr* types and the circulating *S. aureus* lineages. The predominant type, *agr*I, was detected in 39 isolates (39/55, 70.9%) belonging to CC22 (ST22), CC398 (ST398), CC8 (ST72), CC97 (ST97) and CC7 (ST7). The second most frequent *agr* type, *agr*II, was found in 13 isolates (13/55, 23.6%) belonging to CC5 and CC15. *agr*III and *agr*IV were only identified in the two ST1 isolates and one ST121 isolate, respectively. The isolates belonging to the newly described lineages shared the same *agr* type with other STs from the same CC. The exception was the isolate with ST6565 (CC1) that presented *agr*I diverging from ST1, which displayed *agr*II.

The study collection included four pairs of isolates sampled from the same animal (two dogs, one cat and one rabbit), either at distinct times and/or with different colony morphologies (Table S1). Comparing their genotypic and phenotypic characteristics, two of these pairs were indistinguishable by molecular typing, but one corresponded to isolates collected at different times or with distinct morphologies. The remaining pairs of isolates belonged to distinct *Sma*I-PFGE subtypes (Figure 2).

### 2.4. Relationship between Strain Lineage, Host and Antimicrobial Resistance

The relationship between the *S. aureus* clonal lineages detected is depicted in Figure 3. The predominant clonal complex of this collection, CC22 (ST22), corresponded to *S. aureus* that caused SSTIs in dogs, cats, and rabbits. This clone was detected in a dog sample in 2008 and re-emerged in 2012, a time point in which it was established as the predominant clone (Figure 2). Notably, all isolates belonging to this lineage are MRSA and are also resistant to fluoroquinolones. Interestingly, many of the CC22 isolates also harbored heavy-metal resistance genes linked to reduced susceptibility to arsenate and cadmium acetate. The second most frequent clonal complex, CC5, was found throughout the study period (2003–2018) and was mostly associated with SSTI in dogs. This lineage was also linked to reduced susceptibility to cadmium and carriage of the *cadD* gene. In particular, the lineage ST105 (CC5), detected only in two canine isolates in 2018, is associated with MRSA-MDR phenotypes. *S. aureus* of the complex CC398 was isolated from either dogs, cats or rabbits, and presented a variable antibiotic resistance profile. The three clonal lineages associated with CC1 were only detected in canine samples in the last year of the study (2018), mostly without antibiotic resistance. A single ST121 (CC121) isolate was detected in this study related to SSTI in a rabbit. The single isolate collected from a horse belonged to the lineage ST816, which has been sporadically described in the literature.

Overall, throughout the timespan of the study collection and comparing the antibiotic susceptibility profiles of the isolates, we registered an increasing frequency of antimicrobial resistance burden. Despite the disproportionate number of isolates collected between 1999–2009 (*n* = 8) and 2010–2018 (*n* = 47), we can observe that in the first time period, all isolates but one were either fully susceptible or monoresistant to penicillin or fluoroquinolones. A single non-MDR MRSA isolate was recovered in this time period. In opposition, during 2010–2018, only eleven isolates were either fully susceptible or monoresistant to penicillin; the remaining 36 isolates were resistant to two or more classes of antibiotics. The high frequency of MRSA strains in this period (24/47, 51.1%) was a reflection of the dissemination of the ST22 lineage. This increased burden of antibiotic resistance could also be observed within CC5 in the last years of the study (2015–2018).

## 3. Discussion

Bacterial SSTIs are one of the main causes of the attendance of companion animals at veterinary clinics and hospitals [1,2]. Pyoderma is one of the most common skin infections in dogs and cats [3,24], but it is less frequent in horses and rabbits [6]. Bacterial pyoderma is mainly associated with coagulase-positive staphylococci. In dogs, *S. aureus* may be responsible for up to 10% of pyoderma cases [2], whereas in cats, it is often associated with a higher proportion of cases [4,24]. This difference in the frequency rate of *S. aureus* between cats and dogs may reflect the lower adherence of *S. pseudintermedius* to feline corneocytes when compared to canine corneocytes [25]. In our study, *S. aureus* was more associated with canine pyoderma (27 dogs out of 51 animals, 52.9%) than with skin infections in cats (18 cats out of 51 animals, 35.3%), rabbits (4/51, 7.8%) or horses (1/51, 2.0%), eventually reflecting a closer contact between humans and dogs, among other possible reasons.

Skin infections are one of the main causes of antimicrobial prescription in veterinary medicine [1]. The increasing rate of infections caused by MRSA and/or MDR strains has a severe impact on the therapeutic options available and the management of these infections [2]. The recurrent nature of these infections, previous antimicrobial treatment and close contact with humans previously hospitalized are among the risk factors for the occurrence of infections caused by antimicrobial-resistant strains [1,26,27].

Antibiotic resistance was a common feature of this collection of SSTI-related *S. aureus*. Of the 55 isolates studied, only 8 isolates (14.5%) were fully susceptible to the wide panel of antibiotics tested. Eight isolates (14.5%) presented a MDR phenotype, and over half (56.4%) were MRSA.

Some of these isolates were partially characterized in a previous study by Couto and colleagues [12] together with *S. aureus* isolates and other staphylococci related to different infection types in companion animals in Lisbon, Portugal. That previous study documented the increasing trend of MRSA and MDR staphylococcal strains during a 16-year period, with an overall detection of MRSA of over 40% and over 25% for MDR strains [12]. Our study also indicated an increasing trend of staphylococcal resistance to most antimicrobial classes. Although the study collections are not directly comparable, it is interesting to note that we observed a higher MRSA rate (56.4%) as well as a higher frequency of fluoroquinolone resistance (present study: 56.4% vs. 40.7% [12]). On the other hand, a lower rate of MDR phenotypes was found in the current study (present study: 14.5% vs. 25% [12]).

Another survey conducted in Portugal showed that canine bacterial skin infections are generally treated with oral antibiotherapy, particularly amoxicillin-clavulanic acid, cephalexin and fluoroquinolones [28]. Other less frequently prescribed antibiotics include clindamycin, doxycycline, trimethoprim-sulphamethoxazole and minocycline [28]. Topical therapy based on therapeutic baths, skin disinfection and topical antibiotics was also commonly prescribed either alone or in combination [28]. The resistance patterns encountered in our study may reflect the pattern of prescription of oral antibiotics, since resistance to beta-lactams and/or fluoroquinolones was observed in nearly 60% of the isolates, whereas resistance to clindamycin and tetracyclines was registered at lower frequencies (14.5% and 3.6%, respectively). Our data also indicates a significant abundance of arsenate and cadmium resistance determinants, which are linked to a reduced susceptibility toward these antimicrobials. In fact, analysis of MIC distributions detected NWT isolates for arsenate (MIC > 4 mM). For cadmium, although a CO_WT_ could not be established accurately, *cadA* and *cadD* genes were detected in isolates with higher MICs. In previous studies, it had already been observed that *cadA* is associated with high-level resistance to cadmium and low-level resistance to zinc, and *cadD* is associated with low-level resistance to cadmium [29]. The MIC distribution of the remaining heavy metals is similar to that obtained in previous studies in *S. aureus* [29,30,31]. In particular, the study conducted by Kernberger-Fischer and colleagues has proposed cut-off values for zinc chloride (512 mg/L or 3.8 mM) and silver nitrate (32 mg/L or 0.19 mM) [31] that are similar to the ones obtained in the present study, namely 8 mM and 0.125 mM, respectively. In addition, no NWT populations were encountered for these heavy metals in the former study. Heavy metals are used in many topical and hard-surface applications. In particular, compounds with zinc, silver, copper, arsenic and cadmium have been used as antimicrobial agents for several years, both in surface disinfection and in various products as inhibitors/reducing agents of microbial growth [31]. The relative abundance of mobile genetic elements that simultaneously harbor heavy-metal and antibiotic resistance determinants highlights concerns regarding the possible role of heavy metals as a selective factor for the dissemination of antimicrobial resistance [16,32].

The circulating clonal lineages detected in our collection are in accordance with the scarce available literature on companion animals in Portugal. In our collection, the predominant lineage was CC22 (ST22), which accounted for nearly half the isolates, followed by CC5 (ST5, ST105 and ST6535), CC8 (ST72 and ST6566) and CC398 (ST398). In Portugal, some of these lineages have been identified in healthy dogs [33] as well as in diseased dogs, cats and other companion animals [12,34]. MRSA strains were restricted to the lineages CC22 (100.0% of CC22 isolates), CC5 (40% of CC5 isolates), CC8 and CC398 (20% of CC8 and CC398 isolates). Other recent studies in Europe have shown, instead, a similar proportion of several lineages, including CC22, CC5, CC8 and CC398, in MRSA from companion animals [35,36].

The population structure of our animal-associated *S. aureus* collection seems to reflect the predominant lineages circulating in the community and human healthcare settings [37,38,39,40], highlighting the potential sharing of *S. aureus* strains between companion animals and their owners. In fact, several studies have demonstrated a similarity between *S. aureus*/MRSA clones isolated from companion animals, particularly cats and dogs, with those isolated from close-contact humans [35,41,42].

The clonal lineage ST398 has been initially associated with production animals, but its report from companion animals, including cats, dogs and rabbits, is growing [35,43,44]. In fact, the five ST398 isolates identified in this collection were isolated from either dogs, cats or rabbits. The lineage CC1 has been rarely reported in companion animals, with only a few isolates from dogs and cats identified in Austria, France and Germany [35,44,45].

Lineage ST121 is frequently associated with either farm rabbits, wild rabbits or companion rabbits [46,47,48]. In our collection, despite the low number of rabbits analyzed (*n* = 4), only one isolate, dating from 2003, belonged to ST121. The remaining isolates belonged to ST22 or ST398, lineages increasingly reported in rabbits in other studies [49,50,51]. In our collection, the single isolate with equine origin belonged to ST816, which has been reported as a minor clone causing infection in horses [52,53].

## 4. Materials and Methods

### 4.1. Bacterial Isolates

The study collection included 55 *S. aureus* isolated at one university teaching laboratory (Lab 1) and one private diagnostic laboratory (Lab 2), both in Lisbon district, Portugal. These isolates corresponded to all *S. aureus* associated with SSTIs in companion animals recovered during 1999–2018 (Lab 1, *n* = 21) and 2017–2018 (Lab 2, *n* = 34), from dogs (*n* = 27), cats (*n* = 18), rabbits (*n* = 4) and one horse (*n* = 1). Four pairs of isolates were collected from the same animal. The host species was not known for one isolate (Table S1 of the Supplementary Data). Sixteen of these isolates were partially characterized in a previous study [12].

All isolates were grown in tryptic soy broth (TSB) (Oxoid, Hampshire, UK), with shaking or tryptic soy agar (TSA) (Oxoid) at 37 °C.

Identification was confirmed by a species-specific *nuc*-PCR protocol, as described by Poulsen and colleagues [54], with primers described in Table S3 of Supplementary Data.

### 4.2. Antibiotic Susceptibility Testing

Antimicrobial susceptibility was determined for 25 antibiotics by disk diffusion in Mueller-Hinton agar (MHA, Oxoid), according to the recommendations of CLSI for bacteria isolated from animals [18] or EUCAST [20]. Antibiotics discs were obtained from Oxoid or MAST Group (pradofloxacin) (Liverpool, UK). The following antibiotic discs were used: penicillin (PEN, 1 U), cefoxitin (FOX, 30 μg), ciprofloxacin (CIP, 5 μg), enrofloxacin (5 μg), pradofloxacin (5 μg), moxifloxacin (MOX, 5 μg), gentamicin (GEN, 10 μg), kanamycin (KAN, 30 μg), tobramycin (TOB, 10 μg), apramycin (15 μg), amikacin (AMI, 30 μg), tetracycline (TET, 30 μg), minocycline (MIN, 30 μg), tigecycline (TIG, 15 μg), chloramphenicol (CHL, 30 μg), florfenicol (30 μg), erythromycin (ERY, 15 μg), clindamycin (CLI, 2 μg), quinupristin/dalfopristin (QD, 15 μg), linezolid (LIN, 10 μg), trimethoprim-sulfamethoxazole (TRS, 25 μg), rifampicin (RIF, 5 μg), bacitracin (10 U), fusidic acid (FUS, 10 μg), and mupirocin (MUP, 200 μg). Inducible resistance to clindamycin was detected by the D-zone test. The penicillin inhibition zone was examined to detect β-lactamase production. Susceptibility profiles were interpreted according to CLSI VET01S-ED5 for ENR, PRA, TET and CLI [18] or CLSI M100-S32 (bacteria isolated from humans) for PEN, FOX, CIP, MXF, GEN, ERY, QD, MIN, TRS, CHL, LIN, RIF [19] or according to EUCAST for TOB, KAN, AMI, FUS, TIG and MUP [20]. Isolates categorized as intermediate using the CLSI breakpoints were considered resistant. Phenotypic resistance to florfenicol and apramycin was screened by application of the ECOFF values proposed by Costa and colleagues [21].

The reference strains *S. aureus* ATCC25923 and ATCC29213 were used as quality controls. Isolates resistant to one antibiotic of at least three classes of antibiotics were considered MDR [55].

### 4.3. Phenotypic Evaluation of Reduced Susceptibility to Heavy-Metals and Biocides

Susceptibility to the heavy metals zinc chloride (ZnCl_2_), copper sulphate (CuSO_4_·5H_2_0), silver nitrate (AgNO_3_), cadmium acetate ((Cd(CH_3_COO)_2_·2H_2_0) and disodium hydrogen arsenate (Na_2_HAsO_4_·7H_2_0) was evaluated by determination of MICs by the two-fold microdilution method with cation-adjusted Mueller–Hinton broth (CAMHB, Oxoid) as previously described [29]. All reagents were acquired in powder form (Sigma-Aldrich, St. Louis, MO, USA) and dissolved in water. Briefly, from overnight cultures, a cellular suspension equivalent to McFarland 0.5 was prepared in CAMHB and aliquoted in 96-well plates containing two-fold dilutions of the compound to be tested. After incubation at 37 °C for 18 h, the MICs were registered, corresponding to the lowest concentration of heavy metal that inhibited visible bacterial growth.

The analyses of the MIC distributions of heavy metals were used to estimate the presence of non-wild-type (NWT) populations towards these compounds, which are characterized by the presence of acquired resistance mechanism(s) with phenotypic expression [56]. On the contrary, a wild-type (WT) population is considered as not presenting such resistance mechanisms [56]. Both populations can be identified using the cut-off (CO_WT_) value, which corresponds to the highest MIC value encountered in the WT population and is expressed as WT ≤ X mM [56]. Consequently, a NWT population is characterized by MICs > CO_WT_.

The CO_WT_ values were determined using the iterative statistical method (ISM). This method estimates the MIC distribution of the WT population based on a non-linear least squares regression, constructed upon subsets of a log_2_-normal distribution of cumulative counts of MIC data. This analysis allows us to estimate (i) the number of isolates in each subset and (ii) the log_2_ values of the mean MIC of a WT population and associated standard deviation (SD) [57]. The log_2_ values of the mean MIC and SD are used to determine the cut-off value at 99% of the WT population [57]. The ISM was applied using the ECOFFinder datasheet available at http://www.eucast.org/mic_distributions_and_ecoffs/ (accessed on 10 November 2021).

### 4.4. Detection of Resistance Genes by PCR

Total DNA was extracted from each isolate by the boiling method [58]. All isolates were screened for the presence of the resistance genes *mecA* and *blaZ* by PCR. Isolates categorized as resistant or intermediate to antibiotics were also screened for the presence of the genes *erm*(A), *erm*(B), *erm*(C), *msr*(A), *mph*(C), *vga*(A), *vga*(C) (resistance to macrolides, lincosamides, and streptogramins); *tet*(M), *tet*(K), *tet*(L) (resistance to tetracyclines); *aadD*, *aph*(3′)-*IIIa*, *aacA-aphD* (resistance to aminoglycosides); *cat_pC221_*, *fexA* (resistance to phenicols); and *fusB* and *fusC* (resistance to fusidic acid). The collection was also screened for the presence of the heavy-metal resistance genes *cadA*, *cadD* and *arsB.* The control DNAs and primers used are described in Tables S2 and S3 of Supplementary Data.

### 4.5. Molecular Typing

All isolates were characterized by *Sma*I-PFGE as previously described [59]. Macrorestriction profiles were analyzed with the Bionumerics software v 7.6 using the Dice coefficient, and dendrograms were built based on the UPGMA algorithm (1% band tolerance, 0.5% optimization). Isolates with macrorestriction profiles with similarities of ≥80% or ≥97% were assigned to the same PFGE type or subtype, respectively [60]. The genetic diversity of the collection was estimated by calculation of the Simpson’s index of diversity (with a 95% confidence interval), based on *Sma*I-PFGE types [61].

Isolates representative of each *Sma*I-PFGE type were further characterized by MLST according to established protocols [62,63]. Isolates assigned to the same *Sma*I-PFGE type or subtype were considered as belonging to the same ST. Allelic profiles and STs were retrieved from the PubMLST database (accessed on 20 March 2022). New alleles/STs were submitted to PubMLST for validation and allele/ST assignment. Relationships between clonal lineages were inferred with the PHYLOViZ Online freeware using the goeBurst algorithm [64].

*agr* typing was performed for all isolates according to the protocol described by Lina and colleagues [65]. The set of primers used for *agr* typing is described in Table S3.

## 5. Conclusions

This study revealed a genetically diverse *S. aureus* population causing SSTIs in companion animals in Lisbon, Portugal, with evidence of a growing occurrence of CC22 strains, also predominant in human healthcare settings and the community. The finding of a high burden of antimicrobial resistance in these isolates reinforces previous concerns on the rise of resistance to the most-prescribed antibiotics for the management of SSTIs in veterinary medicine. It also raises concerns from a One Health perspective, considering the potential human-animal-human transfer of antimicrobial-resistant *S. aureus*.

## Figures and Tables

**Table 1:** Antibiotic susceptibility profiles and resistance genes for the 55 SSTI-related *S. aureus* isolates studied. Data are only presented for antibiotics with established breakpoints.

Antibiotic	ZD Breakpoint		Number of Isolates (%)			Resistance Determinants (No. Isolates)
	S (mm)	R (mm)	S	I	R	
Penicillin **	≥29 ^b^	≤28 ^b^	10 (18.2%)	-	45 (81.8%)	*blaZ* (45) and *mecA* (31)
Cefoxitin **	≥22	≤21	24 (43.6%)	-	31 (56.4%)	*mecA* (31)
Enrofloxacin *^,a^	≥23	≤16	24 (43.6%)	1 (1.8%)	30 (54.5%)	-
Pradofloxacin *^,a^	≥24	≤19	25 (45.5%)	0 (0%)	30 (54.5%)	-
Ciprofloxacin **	≥21	≤15	24 (43.6%)	1 (1.8%)	30 (54.5%)	-
Moxifloxacin **	≥24	≤20	25 (45.5%)	0 (0%)	30 (54.5%)	-
Erythromycin **	≥23	≤13	47 (85.5%)	0 (0%)	8 (14.5%)	*erm*(C) (4), *erm*(A) (2), n.i. (2)
Clindamycin *^,a^	≥21	≤14	47 (85.5%)	0 (0%)	8 (14.5%) ^c^	*erm*(C) (4), *erm*(A) (2), n.i. (2)
Quinupristin-dalfopristin **	≥19	≤15	55 (100%)	0 (0%)	0 (0%)	-
Tetracycline *^,a^	≥23	≤17	53 (96.3%)	1 (1.8%)	1 (1.8%)	*tet*(K) + *tet*(M) (1), n.i. (1)
Minocycline **	≥19	≤14	54 (98.2%)	1 (1.8%)	0 (0%)	*tet*(K) + *tet*(M) (1)
Tigecycline ***	≥18	<18	55 (100%)	-	0 (0%)	-
Fusidic acid ***	≥24	<24	54 (98.2%)	-	1 (1.8%)	*fusC* (1)
Linezolid **	≥21	≤20	55 (100%)	-	0 (0%)	-
Chloramphenicol **	≥18	≤12	54 (98.2%)	0 (0%)	1 (1.8%)	*fexA* (1)
Trimethoprim-sulfamethoxazole **	≥16	≤10	55 (100%)	0 (0%)	0 (0%)	-
Rifampicin **	≥20	≤16	55 (100%)	0 (0%)	0 (0%)	-
Gentamicin **	≥15	≤12	53 (96.3%)	0 (0%)	2 (3.6%)	*aadD* (1), n.i. (1)
Amikacin ***	≥18	<15	55 (100%)	0 (0%)	0 (0%)	-
Tobramycin ***	≥18	<18	53 (96.3%)	-	2 (3.6%)	*aadD* (1), n.i. (1)
Kanamycin ***	≥18	<18	53 (96.3%)	-	2 (3.6%)	*aadD* (1), n.i. (1)

ZD: zone inhibition diameter; S: susceptible; I: intermediate; R: resistant; * Breakpoint established by CLSI for staphylococci isolated from animals, document VET01S ED5 [18]; ** Breakpoint established by CLSI for staphylococci isolated from humans, document M100-S32 [19]; *** Breakpoint established by EUCAST [20]; ^a^ The breakpoint used is established for isolates of canine and feline origin (enrofloxacin), only canine origin (clindamycin, tetracycline) or only feline origin (pradofloxacin) [18]; ^b^ Isolates with a ZD towards penicillin >29 mm, but with a sharp inhibition border were considered producers of beta-lactamase and thus resistant to penicillin [18]; ^c^ All isolates showed inducible resistance to clindamycin; n.i.: not identified.

**Table 2:** Cut-off (CO_WT_) values of *S. aureus* for the five heavy metals. The CO_WT_ values and estimated wild-type (WT) and non-wild-type (NWT) populations were determined based on the MIC distributions using ECOFFinder.

	CO_WT_	SD (log_2_)	WT Population		NWT Population	
			X ≤ CO_WT_	No. Isolates (%)	X > CO_WT_	No. Isolates (%)
Silver nitrate (AgNO_3_)	0.125 mM	0.48	≤0.125 mM	55 (100%)	>0.125 mM	0 (0%)
Zinc chloride (ZnCl_2_)	8 mM	0.79	≤8 mM	55 (100%)	>8 mM	0 (0%)
Copper sulphate (CuSO_4_)	---	---	---	---	---	---
Cadmium acetate (Cd(CH_3_COO)_2_)	16 mM *	3.20 *	---	---	---	---
Disodium hydrogen arsenate (Na_2_HAsO_4_)	4 mM	0.74	≤4 mM	45 (72.7%)	>4 mM	10 (18.2%)

SD: standard deviation; WT: wild-type; NWT: non-wild-type. * Cut-off values associated with a high SD (>1 log_2_) were not considered accurate.

## Data Availability

All relevant data have been provided in the paper. Raw data can also be provided by the authors upon reasonable request.

## Supplementary Materials

The following are available online at https://www.mdpi.com/article/10.3390/antibiotics11050599/s1, Table S1: *S. aureus* associated with SSTIs in companion animals analyzed in this study; Table S2: Control strains used for the screening of antibiotic resistance; Table S3: Primers used in this study. References [12,23,29,34,40,54,62,63,66,67,68,69,70,71,72,73,74,75,76,77,78,79,80,81,82,83,84] are cited in the supplementary materials.
