# Supplementary material for: Staphylococcus aureus Causing Skin and Soft Tissue Infections in Companion Animals: Antimicrobial Resistance Profiles and Clonal Lineages
Source: Antibiotics (Basel). 2022 Apr 29;11(5):599. doi: 10.3390/antibiotics11050599 (PMC9137735; doi:10.3390/antibiotics11050599)
Supplement: Supplementary file 1 [file antibiotics-11-00599-s001.zip › antibiotics-1685841-supplementary.pdf]

antibiotics

MDPI

Article

# *Staphylococcus aureus* causing skin and soft tissue infections in companion animals: antimicrobial resistance profiles and clonal lineages

**Sofia Santos Costa [1], Rute Ribeiro [1], Maria Serrano [1], Ketlyn Oliveira [1], Carolina Ferreira [1], Marta Leal [1], Constança Pomba [2,3], and Isabel Couto [1,*]**

[1] Global Health and Tropical Medicine, GHTM, Instituto de Higiene e Medicina Tropical, IHMT, Universidade Nova de Lisboa, UNL, Rua da Junqueira 100, 1349-008 Lisboa, Portugal; scosta@ihmt.unl.pt (S.S.C.);
a21000795@ihmt.unl.pt (R.R.); a21000870@ihmt.unl.pt (M.S.); mmm0012@ihmt.unl.pt (K.O.); carolinaf@ihmt.unl.pt (C.F.); a21001253@ihmt.unl.pt (M.L.)

[2] CIISA, Centre of Interdisciplinary Research in Animal Health, Faculty of Veterinary Medicine, University of Lisbon, Avenida da Universidade Técnica, 1300-477 Lisboa, Portugal; cpomba@fmv.ulisboa.pt

[3] GeneVet, Laboratório de Diagnóstico Molecular Veterinário, Rua Quinta da Nora Loja 3B, 2790-140 Carnaxide, Portugal

* Correspondence: icouto@ihmt.unl.pt; Tel.: +351-21-3652652; Fax: +351-21-3632105

**Table S1.** *S. aureus* associated to SSTIs in companion animals analyzed in this study (n = 55).

| Isolate | Biological Sample | Host | Year |
|---|---|---|---|
| **Laboratory 1** | | | |
| **BIOS V4** | Skin exudate | Dog | 2013 |
| **BIOS V5** | Skin exudate | Dog | 2014 |
| **BIOS V6** | Skin exudate | Dog | 2014 |
| **BIOS V19** | Skin exudate | Cat | 2012 |
| **BIOS V20** | Skin exudate | Cat | 2012 |
| **BIOS V21** | Skin exudate | Cat | 2012 |
| **BIOS V22** | Skin exudate | Cat | 2012 |
| **BIOS V30** | Skin exudate | Dog | 2014 |
| **BIOS V31** | Skin exudate | Dog | 2018 |
| **BIOS V60** | Skin exudate | Horse | 2001 |
| **BIOS V61** | Skin exudate | Dog | 2003 |
| **BIOS V62** | Skin exudate | Cat | 2005 |
| **BIOS V63** | Paw area exudate | Dog | 2008 |
| **BIOS V70** | Skin exudate | Dog | 2015 |
| **BIOS V74[A]** | Pustule liquid | Dog | 2003 |
| **BIOS V75** | Skin exudate | Dog | 2005 |
| **BIOS V76** | Skin exudate | Cat | 2012 |
| **BIOS V85[A]** | Pustule liquid | Dog | 2003 |
| **BIOS V118** | Skin exudate | Dog | 2007 |
| **BIOS V128** | Skin exudate | Cat | 2018 |
| **BIOS V129** | Skin exudate | Rabbit | 2003 |

(A - D): Isolates collected from the same animal.

**Table S1.** (cont.) *S. aureus* associated to SSTIs in companion animals analyzed in this study (n = 55).

| Isolate | Biological Sample | Host | Year |
|---|---|---|---|
| **Laboratory 2** | | | |
| **BIOS V147** | Skin exudate | Rabbit | 2017 |
| **BIOS V150** | Skin exudate | Dog | 2017 |
| **BIOS V151** | Skin exudate | Cat | 2017 |
| **BIOS V153** | Skin exudate | Dog | 2018 |
| **BIOS V154** | Skin exudate | Cat | 2018 |
| **BIOS V155[B]** | Skin exudate | Cat | 2018 |
| **BIOS V156** | Skin exudate | Dog | 2018 |
| **BIOS V157** | Skin exudate | Dog | 2018 |
| **BIOS V158** | Skin exudate | Cat | 2017 |
| **BIOS V159** | Skin exudate | Cat | 2018 |
| **BIOS V160** | Skin exudate | Cat | 2018 |
| **BIOS V161** | Skin exudate | Rabbit | 2018 |
| **BIOS V168** | Skin exudate | Cat | 2018 |
| **BIOS V172** | Skin exudate | Dog | 2018 |
| **BIOS V178** | Skin exudate | Cat | 2018 |
| **BIOS V183** | Skin exudate | Dog | 2017 |
| **BIOS V184[B]** | Skin exudate | Cat | 2018 |
| **BIOS V185** | Skin exudate | Dog | 2018 |
| **BIOS V186** | Skin exudate | Dog | 2018 |
| **BIOS V187** | Skin exudate | Dog | 2018 |
| **BIOS V200[C]** | Skin exudate | Dog | 2018 |
| **BIOS V201[C]** | Skin exudate | Dog | 2018 |
| **BIOS V202** | Skin exudate | Cat | 2018 |
| **BIOS V203** | Skin exudate | Dog | 2018 |
| **BIOS V204[D]** | Skin exudate | Rabbit | 2017 |
| **BIOS V245** | Skin exudate | Dog | 2018 |
| **BIOS V250** | Skin exudate | Dog | 2018 |
| **BIOS V255** | Skin exudate | Dog | 2018 |
| **BIOS V257** | Skin exudate | Cat | 2017 |
| **BIOS V258[D]** | Skin exudate | Rabbit | 2017 |
| **BIOS V279** | Skin exudate | Cat | 2017 |
| **BIOS V295** | Skin exudate | Unknown | 2018 |
| **BIOS V296** | Skin exudate | Dog | 2018 |
| **BIOS V300** | Skin exudate | Dog | 2018 |

(A - D): Isolates collected from the same animal.

**Table S2.** Control strains used for the screening of antibiotic resistance genes.

| Target Gene | Control strain | Reference |
|---|---|---|
| ***mecA*** | *S. aureus* SM1 | [66] |
| ***blaZ*** | *S. aureus* SM1 | [66] |
| ***erm*(A)** | *S. aureus* SM39 | [29] |
| ***erm*(B)** | *S. pseudintermedius* 4877/10 | [12] |
| ***erm*(C)** | *S. aureus* SM26 | [29] |
| ***msr*(A)** | *S. epidermidis* FMV-51 | [67] |
| ***mph*(C)** | *S. epidermidis* FMV-51 | [67] |
| ***vga*(A)** | *S. epidermidis* FMV-51 | [67] |
| ***vga*(C)** | *S. aureus* 49.1 | [34] |
| ***fusB*** | *S. epidermidis* FMV-97 | [68] |
| ***fusC*** | *S. epidermidis* FMV-34 | [68] |
| ***tet*(K)** | *S. epidermidis* ATCC12228 | [69] |
| ***tet*(M)** | *S. aureus* H4/09 | [34] |
| ***tet*(L)** | *S. aureus* 25.1 | [34] |
| **A07** | *S. aureus* 25.1 | [34] |
| **C01** | *S. aureus* 25.1 | [34] |

**Table S3.** Primers used in this study.

| Target Gene | Primers | Nucleotide Sequence (5′-3′) | Amplicon Size (bp) | Reference |
| --- | --- | --- | --- | --- |
| ***S. aureus* identification** | | | | |
| ***nuc*** | nuc-Fw | TCAGCAAATGCATCACAAACAG | 255 | [54] |
| | nuc-Rv | CGTAAATGCACTTGCTTCAGG | | |
| **Screening of resistance genes and mutations** | | | | |
| ***mecA*** | mecA_Fw | GGTCCCATTAACTCTGAAG | 1040 | [70] |
| | mecA_Rv | AGTTCTGCAGTACCGGATTTGC | | |
| ***blaZ*** | blaZ_Fw | GATAAGAGATTTGCCTATGC | 533 | [71] |
| | blaZ_Rv | GCATATGTTATTGCTTGACC | | |
| ***erm*(A)** | erm(A)_Fw | AAGCGGTAAACCCCTCTGAG | 442 | [72] |
| | erm(A)_Rv | TCAAAGCCTGTCGGAATTGG | | |
| ***erm*(B)** | erm(B)_Fw | TGGAACAGGTAAAGGGCATT | 433 | [29] |
| | erm(B)_Rv | TGTGGTATGGCGGGTAAGTT | | |
| ***erm*(C)** | erm(C)_Fw | TCGTAACTGCCATTGAAATA | 348 | [29] |
| | erm(C)_Rv | TCACTTTAGGTTTAGGATGAAA | | |
| ***msr*(A)** | msr(A)_Fw | GATTGTCCCAAGCCAGTAAA | 445 | [40] |
| | msr(A)_Rv | GCCATTTGCACTTTAGGAGA | | |
| ***mph*(C)** | mph(C)_Fw | ATGACTCGACATAATGAAAT | 900 | [73] |
| | mph(C)_Rv | CTACTCTTTCATACCTAACTC | | |
| ***vga*(A)** | vga(A)_Fw | ACCCGAGACATCTTCACCAC | 400 | [34] |
| | vga(A)_Rv | GGAAATTGACGAGGGGAGA | | |
| ***vga*(C)** | vga(C)_Fw | ACGAATAAAGGGATCGAAGC | 510 | [40] |
| | vga(C)_Rv | AGCACATGCACAGGTTTGTA | | |
| ***fusB*** | fusB_Fw | ATTCAATCGGAAACCTATAATGATA | 292 | [74] |
| | fusB_Rv | TTATATATTTCCGATTTGATGCAAG | | |
| ***fusC*** | fusC_Fw | GATATTGATATCTCGGACTT | 128 | [75] |
| | fusC_Rv | AGTTGACTTGATGAAGGTAT | | |
| ***tet*(K)** | tet(K)_Fw | GTAGCGACAATAGGTAATAGT | 361 | [76] |
| | tet(K)_Rv | GTAGTGACAATAAACCTCCTA | | |
| ***tet*(M)** | tet(M)_Fw | GTTAAATAGTGTTCTTGGAG | 657 | [77] |
| | tet(M)_Rv | CTAAGATATGGCTCTAACAA | | |
| ***tet*(L)** | tet(L)_Fw | GTCGGTAATTGGGTTTGTTG | 421 | [78] |
| | tet(L)_Rv | TGACAGCACGCTAACGATAA | | |
| ***fexA*** | fexA_Fw | GTACTTGTAGGTGCAATTAGGCTGA | 1272 | [79] |
| | fexA_Rv | CGCATCTGAGTAGGACATAGCGTC | | |
| ***cat*(pC221)** | catpC221_Fw | ATTTATGCAATTATGGAAGTTG | 435 | [73] |
| | catpC221_Rv | TGAAGCATGGTAACCATCAC | | |
| ***aph*(3′)*-IIIa*** | aph3-III_Fw | CCGCTGCGTAAAAGATAC | 609 | [80] |
| | aph3-III_Rv | GTCATACCACTTGTCCGC | | |
| ***aacA-aphD*** | aacA-aphD_Fw | CAGAGCCTTGGGAAGATGAAG | 348 | [81] |
| | aacA-aphD_Rv | CCTCGTGTAATTCATGTTCTGGC | | |

**bp:** base pair; **Fw:** "forward"; **Rv:** "reverse"

**Table S3.** (cont.) Primers used in this study.

| Target Gene | Primers | Nucleotide Sequence (5′-3′) | Amplicon Size (bp) | Reference |
|---|---|---|---|---|
| ***aadD*** | aadD_Fw | GGAAGCAGAGTTCAGCCATG | 266 | [82] |
| | aadD_Rv | TGCCTGCATATTCAAACAGC | | |
| **Screening of biocide and heavy metal resistance genes** | | | | |
| ***qacA/B*** | qacA/B_Fw | GCTGCATTTATGACAATGTTTG | 628 | [83] |
| | qacA/B_Rv | AATCCCACCTACTAAAGCAG | | |
| ***smr*** | Smr_Fw | ATAAGTACTGAAGTTATTGGAAGT | 285 | [84] |
| | Smr_Rv | TTCCGAAAATGTTTAACGAAACTA | | |
| ***qacG*** | qacG_Fw | CAACAGAAATAATCGGAACT | 275 | [67] |
| | qacG_Rv | TACATTTAAGAGCACTACA | | |
| ***qacJ*** | qacJ_Fw | CTTATATTTAGTAATAGC | 306 | [67] |
| | qacJ_Rv | GATCCAAAAACGTTAAGA | | |
| ***arsB*** | arsB_Fw | GCGAGTTTATCCAAATTCCTG | 476 | [67] |
| | arsB_Rv | CCACGAAATCTTCACACCTTT | | |
| ***cadA*** | cadA_Fw | CCAAAGCGGTAGATGACGAA | 553 | [29] |
| | cadA_Rv | GCAAGTGGATGTTGTGAACG | | |
| ***cadD*** | cadD_Fw | GGTGTATTATGATTCAAACGGT | 614 | [29] |
| | cadD_Rv | TCCTAAAATTGTTTGAATAGTG | | |
| **Identification of ST398** | | | | |
| **A07** | A07_Fw | GATCCCAGAATACTTAAATA | 197 | [23] |
| | A07_Rv | TGACCGTAATCTTGTAAATA | | |
| **C01** | C01_Fw | CATTCATCACACGTATATTC | 140 | |
| | C01_Rv | GGTGATTATTCATGGTTAAG | | |
| **MLST typing** | | | | |
| ***arcC*** | arcC_Fw | CCTTTATTTGATTCACCAGCG | 577 | [63] |
| | arcC_Rv | AGGTATCTGCTTCAATCAGCG | | |
| ***aroE*** | aroE_Fw | ATCGGAAATCCTATTTCACATTC | 536 | |
| | aroE_Rv | GGTGTTGTATTAATAACGATATC | | |
| ***glpF*** | glpF_Fw | CTAGGAACTGCAATCTTAATCC | 576 | |
| | glpF_Rv | TGGTAAAATCGCATGTCCAATTC | | |
| ***gmk*** | gmk_Fw | ATCGTTTTATCGGGACCATC | 488 | [62] |
| | gmk_Rv | TCATTAACTACAACGTAATCGTA | | |
| ***pta*** | pta_Fw | GTTAAAATCGTATTACCTGAAGG | 575 | |
| | pta_Rv | GACCCTTTTGTTGAAAAGCTTAA | | |
| ***tpi*** | tpi_Fw | TCGTTCATTCTGAACGTCGTGAA | 475 | |
| | tpi_Rv | TTTGCACCTTCTAACAATTGTAC | | |
| ***yqiL*** | yqiL_Fw | CAGCATACAGGACACCTATTGGC | 598 | |
| | yqiL_Rv | CGTTGAGGAATCGATACTGGAAC | | |

**bp:** base pair; **Fw:** "forward"; **Rv:** "reverse"

## References


12. Couto, N.; Monchique, C.; Belas, A.; Marques, C.; Gama, L.T.; Pomba, C. Trends and molecular mechanisms of antimicrobial resistance in clinical staphylococci isolated from companion animals over a 16 year period. *J. Antimicrob. Chemother.* **2016,** *71*, 1479-1487. doi: 10.1093/jac/dkw029.
23. van Wamel, W. J. B.; Maňásková, S.H.; Fluit, A. C.; Verbrugh, H.; de Neeling, A. J.; van Duijkeren, E.; van Belkum, A. Short term micro-evolution and PCR-detection of methicillin-resistant and -susceptible *Staphylococcus aureus* sequence type 398. *Eur. J. Clin. Microbiol. Infect. Dis.* **2010**, *29*, 119– 122. doi: 10.1007/s10096-009-0816-3
29. Costa, S.S.; Palma, C.; Kladec, K.; Fessler, A.T.; Viveiros, M.; Melo-Cristino, J.; Schwarz, S.; Couto, I. Plasmid-borne antimicrobial resistance of *Staphylococcus aureus* isolated in a hospital in Lisbon, Portugal. *Microb Drug Resist.* **2016**, *22*, 617-626. doi: 10.1089/mdr.2015.0352
34. Couto, N.; Belas, A.; Kadlec, K.; Schwarz, S.; Pomba, C. Clonal diversity, virulence patterns and antimicrobial and biocide susceptibility among human, animal and environmental MRSA in Portugal. *J. Antimicrob. Chemother.* **2015**, *71*, 1479-1487. doi: 10.1093/jac/dkv141
40. Ferreira, C.; Costa, S.S.; Serrano, M.; Oliveira, K.; Trigueiro, G.; Pomba, C.; Couto, I. Clonal Lineages, Antimicrobial Resistance, and PVL Carriage of *Staphylococcus aureus* associated to skin and soft-tissue infections from ambulatory patients in Portugal. *Antibiotics* **2021**, *10*, 345. https://doi.org/10.3390/antibiotics10040345
54. Poulsen, A.B.; Skov, R.; Pallesen, L.V. Detection of methicillin resistance in coagulase-negative staphylococci and in staphylococci directly from simulated blood cultures using the EVIGENE MRSA Detection Kit. *J. Antimicrob. Chemother.* **2003**, *51*, 419-421. doi: 10.1093/jac/dkg084
62. Enright, M. C.; Day, N. P.; Davies, C. E.; Peacock, S. J.; Spratt, B. G. Multilocus sequence typing for characterization of methicillin-resistant and methicillin-susceptible clones of *Staphylococcus aureus*. *J. Clin. Microbiol.* **2000**, *38*, 1008–1015. doi: 10.1128/JCM.38.3.1008-1015.2000
63. Crisóstomo, M. I.; Westh, H.; Tomasz, A.; Chung, M.; Oliveira, D. C.; de Lencastre, H. The evolution of methicillin resistance in *Staphylococcus aureus*: similarity of genetic backgrounds in historically early methicillin-susceptible and -resistant isolates and contemporary epidemic clones. *Proc. Natl. Acad. Sci.* **2001**, *98*, 9865–9870. doi: 10.1073/pnas.161272898
66. Costa, S.S.; Falcão, C.; Viveiros, M.; Machado, D.; Martins, M.; Melo-Cristino, J.; Amaral, L.; Couto, I. Exploring the contribution of efflux on the resistance to fluoroquinolones in clinical isolates of *Staphylococcus aureus*. BMC Microbiology **2011**, *11*, 241. doi: 10.1186/1471-2180-11-241
67. Holtreman, F. D. Characterization of plasmids of *Staphylococcus epidermidis* and correlation with efflux-mediated resistance. 2018. MSc Thesis in Biomedical Sciences, Universidade NOVA de Lisboa. http://hdl.handle.net/10362/53495
68. Rosa, M.S.R. O. Contribution of efflux to antimicrobial resistance in *Staphylococcus epidermidis*. **2017**. MSc Thesis in Biomedical Sciences, Universidade NOVA de Lisboa. http://hdl.handle.net/10362/20443
69. MacLea, K.; Trachtenberg, A. Complete genome sequence of *Staphylococcus epidermidis* ATCC 12228 chromosome and plasmids, generated by long-read sequencing. *Genome Announc*. **2017**, *5*, e00954-17. doi: 10.1128/genomeA.00954-17
70. Petinaki, E.; Arvaniti, A.; Dimitracopoulos, G.; Spiliopoulou, I. Detection of *mecA*, *mecR1* and *mecI* genes among clinical isolates of methicillin-resistant staphylococci by combined polymerase chain reactions. *J. Antimicrob. Chemother.* **2001**, *47*, 297-304. doi: 10.1093/jac/47.3.297
71. Milheiriço, C.; Portelinha, A.; Krippahl, L.; de Lencastre, H.; Oliveira, D.C. Evidence for a purifying selection acting on the β-lactamase locus in epidemic clones of methicillin-resistant *Staphylococcus aureus*. *BMC Microbiol.* **2011,** *11*, 76. doi: 10.1186/1471-2180-11-76
72. Jensen, L.B.; Hammerum, A.M.; Bager, F.; Aarestrup, F.M. Streptogramin resistance among *Enterococcus faecium* isolated from production animals in Denmark in 1997. *Microb. Drug Resist.* **2002**, *8*, 369-374. doi: 10.1089/10766290260469642
73. Schnellmann, C.; Gerber, V.; Rossano, A.; Jaquier, V.; Panchaud, Y.; Doherr, M.G.; Thomann, A.; Straub, R.; Perreten, V. Presence of new *mecA* and *mph*(C) variants conferring antibiotic resistance in *Staphylococcus* spp. isolated from the skin of horses before and after clinic admission. *J. Clin. Microbiol.* **2006**, *44*, 4444-4454. doi: 10.1128/JCM.00868-06
74. O'Neill, A.J.; Larsen, A.R.; Henriksen, A.S.; Chopra, I. A fusidic acid-resistant epidemic strain of *Staphylococcus aureus* carries the *fusB* determinant, whereas *fusA* mutations are prevalent in other resistant isolates. *Antimicrob. Agents Chemother.* **2004**, *48*, 3594-3597. doi: 10.1128/AAC.48.9.3594-3597.2004
75. Castanheira, M.; Watters, A.A.; Bell, J.M.; Turnidge, J.; Jones, R.N. Fusidic acid resistance rates and prevalence of resistance mechanisms among *Staphylococcus spp*. isolated in North America and Australia, 2007-2008. *Antimicrob. Agents Chemother.* **2010**, *54*, 3614-3617. doi: 10.1128/AAC.01390-09
76. Strommenger, B.; Kettlitz, C.; Werner, G.; Witte, W. Multiplex PCR assay for simultaneous detection of nine clinically relevant antibiotic resistance genes in *Staphylococcus aureus*. *J. Clin. Microbiol.* **2003**, *41*, 4089-4094. doi: 10.1128/jcm.41.9.4089-4094.2003.

77. Aarestrup, F.; Agerso, Y.; Gerner–Smidt, P.; Madsen, M.; Jensen, L. Comparison of antimicrobial resistance phenotypes and resistance genes in *Enterococcus faecalis* and *Enterococcus faecium* from humans in the community, broilers, and pigs in Denmark. *Diagn. Microbiol. Infect. Dis.* **2000**, *37*, 127-137. doi: 10.1016/s0732-8893(00)00130-9.
78. Costa, S.; Oliveira, V.; Serrano, M.; Pomba, C.; Couto, I. Phenotypic and Molecular Traits of *Staphylococcus coagulans* Associated with Canine Skin Infections in Portugal. *J. Antibiot.* **2021**, *10*, 1-15. doi:10.3390/antibiotics10050518
79. Kehrenberg, C.; Schwarz, S. Florfenicol-chloramphenicol exporter gene *fexA* is part of the novel transposon Tn558. *Antimicrob. Agents Chemother.* **2005**, *49*,813-815. doi: 10.1128/AAC.49.2.813-815.2005
80. Perreten, V.; Vorlet-Fawer, L.; Slickers, P.; Ehricht, R.; Kuhnert, P.; Frey, J. Microarray-based detection of 90 antibiotic resistance genes of gram-positive bacteria. *J. Clin. Microbiol.* **2005**, *43*, 2291-2302. doi: 10.1128/JCM.43.5.2291-2302.2005
81. Vakulenko, S.B.; Donabedian, S.M.; Voskresenskiy, A.M.; Zervos, M.J.; Lerner, S.A.; Chow, J.W. Multiplex PCR for detection of aminoglycoside resistance genes in enterococci. *Antimicrob. Agents Chemother.* **2003**, *47*, 1423-1426. doi: 10.1128/AAC.47.4.1423-1426.2003
82. Kobayashi, N.; Alam, M.; Nishimoto, Y.; Urasawa, S.; Uehara, N.; Watanabe, N. Distribution of aminoglycoside resistance genes in recent clinical isolates of *Enterococcus faecalis*, *Enterococcus faecium* and *Enterococcus avium*. *Epidemiol. Infect.* **2001**,*126*, 197-204. doi: 10.1017/s0950268801005271
83. Anthonisen, I. L.; Sunde, M.; Steinum, T. M.; Sidhu, M. S.; Sorum, H. Organization of the antiseptic resistance gene *qacA* and Tn552-related beta-lactamase genes in multidrug- resistant *Staphylococcus haemolyticus* strains of animal and human origins. *Antimicrob. Agents Chemother*. **2002**, *46*, 3606-3612. doi: 10.1128/AAC.46.11.3606-3612.2002
84. Bjorland, J.; Sunde, M.; Waage, S. Plasmid-borne *smr* gene causes resistance to quaternary ammonium compounds in bovine *Staphylococcus aureus*. *J. Clin. Microbiol*. **2001**, *39*, 3999-4004. doi: 10.1128/JCM.39.11.3999-4004.2001
